# Improving Medication Safety in Chronic Kidney Disease Using Rule-Based and Artificial Intelligence–Based Clinical Decision Support Systems: A Systematic Review of Randomized Controlled Trials

**DOI:** 10.12688/f1000research.178438.2

**Published:** 2026-08-28

**Authors:** Asrul Ismail, Rani Sauriasari, Arry Yanuar, Dodi Sudiana, Fonny Cokro

**Affiliations:** 1 Clinical and Social Pharmacy Laboratory, Faculty of Pharmacy, Universitas Indonesia, Depok, West Java, 16424, Indonesia; 2 Health Innovation Study and Policy Research Group, Faculty of Pharmacy, Universitas Indonesia, Depok, West Java, 16424, Indonesia; 3 Biomedical Computation and Drug Design Research Group, Faculty of Pharmacy, Universitas Indonesia, Depok, West Java, 16424, Indonesia; 4 Department of Electrical Engineering, Faculty of Engineering, Universitas Indonesia, Kampus UI, Depok, West Java, 16424, Indonesia; 5 Artificial Intelligence and Data Engineering Research Center (AIDE-RC), Faculty of Engineering, Universitas Indonesia, Kampus UI, Depok, West Java, 16424, Indonesia; 6 Department of Pharmacy, School of Medicine and Health Sciences, Atma Jaya Catholic University, South Jakarta, DKI Jakarta, 12930, Indonesia

**Keywords:** clinical decision support systems, chronic kidney disease, medication safety, renal dose adjustment, artificial intelligence, randomized controlled trials

## Abstract

**Background:**

Optimization of renal drug dosing to avoid drug toxicity is essential in Chronic Kidney Disease (CKD), yet prescribing errors are common. CDSS with rule-based and AI/ML based tools are used to address this safety gap; however, their impact remains uncertain.

**Methods:**

We performed a PRISMA-guided systematic review and meta-analysis of RCTs comparing rule-based or AI/ML CDSS with usual care comparators among adults with CKD or at risk of CKD-related prescribing errors. The primary outcome was a medication safety endpoint aligned with the CDSS logic (appropriate renal dosing, potentially inappropriate prescribing, and medication errors). To address heterogeneity, we supplemented meta-analysis with a structured Best Evidence Synthesis and trial-level mapping by delivery mode and workflow stage.

**Results:**

Among 20 RCTs meeting inclusion criteria, 6 provided meta-analytic data. Pooled across four trials, CDSS improved appropriate renal dosing (RR 1.76; 95% CI 1.13–2.74), but heterogeneity was extreme (I ^2^ = 97%) and the 95% prediction interval (0.75–4.14) crossed the null; the pooled estimate is therefore a context-dependent average rather than a transportable effect, and benefit cannot be assured in a new setting. A consistent direction of effect favoring CDSS came instead from the Best Evidence Synthesis. Documentation of CKD in electronic health records improved consistently (RR 1.19; 95% CI 1.07–1.32; I ^2^ = 0%). Current evidence was predominantly interruptive order-entry interventions; clinician compliance ranged 17–74% owing to alert fatigue, time constraints, and unclear system function and override processes.

## 1. Introduction

Chronic kidney disease (CKD) is an important global health issue leading to high incidence of morbidity, mortality, and utilization of healthcare resources. The complex and often multi-morbidity burden with which CKD patients present, combined with the polypharmacy regimens that they are commonly exposed to, may render them vulnerable to harm from medications. Renal function–based medication prescribing, periodic checks, and timely treatment changes are required to ensure optimal management, prevent adverse outcomes, and delay disease progression. ^1– 6^ In practice, however, decision-making on dosing and monitoring may occur under extreme time pressure, and clinicians may struggle with the increasing complexity of electronic health records (EHRs) that obscure key laboratory, diagnostic, and medication information at the point of care. ^7, 8^ In clinical nephrology, these limitations are further exacerbated in nephrology practice, especially in patients receiving renally cleared medications, experiencing nephrotoxic exposures, and receiving antihypertensive treatments. In such high-risk environments, even small errors in prescribing can lead to an acute kidney injury or drive unnecessary disease progression. These phenomena perpetuate a persistent gap between evidence-based recommendations and actual care, particularly for high-risk prescribing decisions, and identify medication safety in CKD as a priority domain for quality improvement initiatives/programs and patient safety efforts.

Clinical decision support systems (CDSS) have emerged as a key strategy to close this gap. ^9– 13^ Broadly, current CDSS follow two trajectories. Rule-based systems encode guideline logic and expert rules into deterministic alerts or order sets, typically triggered by thresholds in kidney function, prescribed drugs, or other structured data. ^14, 15^ More recently, artificial intelligence–enabled CDSS (AI-CDSS) have been developed that use longitudinal data and machine-learning models to generate individualized risk predictions or treatment recommendations. ^16– 18^ Both approaches aim to standardize care processes, support safer prescribing, and reduce preventable harm without unduly increasing clinicians’ cognitive workload. From a translational perspective, renal CDSS operationalize evidence into real-time decisions at the point of care. However, in practice, CDSS activities are deployed on a variety of platforms (e.g., EHR/CPOE-integrated, Web-based, mobile or standalone tools) and embedded within different workflow stages (e.g., prescribing vs monitoring) which may have important implications for uptake, usability, and effectiveness. Therefore, by investigating how trials correspond to implementation configurations and outcome categories we can also seek to understand where is the evidence base well-developed and where downstream evaluation remains weak.

While more widely used, the overall effect of CDSS on the quality and safety of care in CKD remains unclear. Prior reviews of CDSS have often pooled heterogeneous conditions and interventions, making it difficult to determine where CDSS provide the greatest value for CKD specifically. ^19– 22^ Moreover, the majority of measurements are primarily based on proximal process measures (e.g., renal dosing, CKD documentation, or guideline adherence) and do not have a direct correlation with downstream patient-safety outcomes such as adverse drug events, hospitalization, and progression to end-stage kidney disease.

Topics on how to practice and their relation to safety are inconsistently reported and inadequately aggregated. ^23– 26^ The primary obstacle to overcoming CDSS implementation in nephrology is no longer data capture but understanding which system designs will affect real clinical outcomes (both kidney-disease progression and dialysis-related complications). Answering this question is important to avoid decision support tools remaining mere documentation tools. These evidence gaps indicate that using only meta-analytic summary estimates is inadequate for informing clinical policy or directing future research in renal drug safety. Instead, a linchpin is an overarching model that transparently relates the functionality of CDSS and workflow integration to actual care processes. The dissociation of well-controlled improvement in administrative notes and the widely varying dosing errors once again points to an entrenched environmental dependence. If this context-dependence is disregarded, there is a danger of turning towards statistical abstractions that only hide the actual determinants of success.

## 2. Methods

### 2.1 Study registration

This review followed PRISMA guidelines (Preferred Reporting Items for Systematic Reviews and Meta-Analyses) and The Cochrane Handbook for Systematic Reviews and Meta-Analyses. ^27, 28^ This review was registered on the PROSPERO website (Registration No. CRD420251139903).

### 2.2 Search strategy and selection criteria

Systematic searches of PubMed, Scopus, ScienceDirect, and ProQuest were conducted for each database through August 2025. Medical Subject Headings (MeSH) and a combination of free-text words were used in the search, without any language limitation. The detailed search strategy is available as Extended data (Appendix 1: Search strategy).

Eligible studies were restricted to RCTs examining digital CDSS applied for clinical decision-making in CKD, including EHR/CPOE-interfaced solutions and web-based or mobile (standalone) electronic decision supports. The interventions might be assisted by AI/ML or rule-based. Trials had to have at least one process domain concerning medication safety. The primary outcome was appropriate dosing according to renal function, and secondary outcomes included CKD identification/documentation, death rate, renal function (as measured by serum creatinine/eGFR), BP/CV events and usability of the CDSS as well as implementation outcomes such as adherence to or compliance with recommendations made by the CDSS when reported. Non-randomized studies, non-electronic databases, and those not specifically for CKD were excluded. Details are available in Tables 1 and 2.

**Table 1.** Eligibility criteria (PICOS-aligned).

Domain	Inclusion	Key exclusions
Population	Chronic Kidney Disease (CKD) cohorts in routine care (adult CKD stages as applicable).	Pediatric-only studies (unless adults predominated).
Intervention	Digital Clinical Decision Support Systems (CDSS) used for clinical decision-making (including EHR/CPOE-integrated, web-based, mobile, or standalone tools): AI/ML-based or rule-based. Must target ≥1 of: treatment advice; renal function–guided dosing; CKD identification/documentation; laboratory monitoring; nephrology referral; other management supports.	Non-digital/paper-only decision aids.
Comparator	Usual/standard care (paper/EHR without CDSS) or alternative non-AI/rule configurations, as reported.	—
Outcomes (eligibility scope)	At least one relevant process or clinical endpoint within the scope above.	Protocols with no clinical outcomes; editorials/commentaries.
Study design	Randomized Controlled Trials (individual, cluster, or crossover).	Non-randomized/observational designs.

Abbreviations: CDSS = Clinical Decision Support System(s); AI/ML = Artificial Intelligence/Machine Learning; CPOE = Computerized Provider Order Entry; EHR = Electronic Health Record; CKD = Chronic Kidney Disease; RCT = Randomized Controlled Trial; PICOS = Population, Intervention, Comparator, Outcomes, Study design.

**Table 2.** Outcomes and operationalization.

Outcome category	Operational definition (analysis unit)	Effect metric	Notes/handling
Primary: Appropriate renal dosing	Proportion of medication instances or patient–drug dispensings concordant with renal-function guidelines.	Risk Ratio (RR)	When only “inappropriate/excess dosing” or “medication error” was reported, appropriate counts were arithmetically derived without changing denominators.
CKD recognition (EHR)	Presence of CKD diagnosis/problem list consistent with clinical criteria (patient-level).	RR	Structured EHR lists/codes.
Mortality	All-cause or specified mortality (patient-level).	RR or HR (time-to-event)	Prefer HR via generic inverse variance when available; do not pool HR with RR.
Renal function	eGFR level/slope, creatinine (patient-level).	MD/SMD	Harmonize units; prespecify directionality.
Blood pressure/CV events	SBP/DBP; cardiovascular events (patient-level).	MD/SMD; RR	As reported.
Usability/acceptability	SUS scores, adoption/override, time-to-action (latency).	MD	Report implementation metrics narratively if heterogeneous.
Guideline adherence	ACEi/ARB prescribing; nephrology referral; ESA adherence (patient-level).	RR	Define denominator clearly (eligible patients/orders).

Abbreviations: CKD = Chronic Kidney Disease; EHR = Electronic Health Record; eGFR = estimated Glomerular Filtration Rate; SBP = Systolic Blood Pressure; DBP = Diastolic Blood Pressure; CV = Cardiovascular; SUS = System Usability Scale; HR = Hazard Ratio; RR = Risk Ratio; MD = Mean Difference; SMD = Standardized Mean Difference; ESA = Erythropoiesis-Stimulating Agent; ACEi = Angiotensin-Converting Enzyme inhibitor; ARB = Angiotensin Receptor Blocker.

### 2.3 Data extraction

Data were imported into Covidence and deduplicated, where data were also screened (titles/abstracts) with full texts reviewed for eligibility. A standard Excel form was used to record study features (title/authors/year, setting/country, RCT design/participants), CDSS features (functionality/classifications/intervention(s)), comparator(s), outcomes (primary/secondary), data types, and arm-specific sample sizes/follow-up. Screening and data extraction were performed by two independent reviewers, with a third reviewer to resolve any disagreements.

### 2.4 Risk of bias in included studies

Risk of bias across studies was assessed with Cochrane’s RoB 2 for the following five domains: randomization process, deviations from intended interventions, missing outcome data, measurement of outcome, and selection of reported results. Risk of bias (RoB) judgements were performed using the RoB 2 guidance: if all domains were at low risk, then the overall RoB was classified as low; if one or more domains were judged to be high, the overall judgement was high; otherwise, some concerns. Methodological quality was assessed by two reviewers working independently of one another, and disagreements were resolved by discussion or a third reviewer.

### 2.5 Reporting bias assessment

We did not formally assess publication bias (e.g., funnel plot–based methods) because the number of studies contributing to each synthesis was limited and the included trials were highly heterogeneous in interventions and outcomes.

### 2.6 Synthesis methods

Meta-analyses were performed using RevMan 5.2 (accessed September 2025) with fixed- and random-effects models. Dichotomous results were aggregated as risk ratios using the Mantel–Haenszel test; continuous ones were calculated as MD (or SMD). We used fixed effects for low heterogeneity (I ^2^ ≤ 50% and conceptually homogeneous outcomes) and random effects models for high heterogeneity (I ^2^ > 50%) or important design/setting variation. For uncertainty, 95% CIs were provided; for heterogeneity, I ^2^ and Chi ^2^. A 0.5 continuity correction was applied to zero cells when necessary, and multi-arm studies were combined as per the Cochrane Handbook to prevent double-counting. Due to the substantial heterogeneity we found in our main effects, we also presented pooled effect estimates with prediction intervals (PI) for a better appreciation of expected results across various clinical settings. As the pooled estimates themselves provide limited refinement of information, due to how implementation is nuanced and varied at each site, our meta-analysis was also given support with a structured Best Evidence Synthesis (BES) and trial-level mapping by delivery mode and workflow stage, incorporating TIDieR elements; trial network mapping was performed using Cytoscape (version 3.10.4). Quantitative pooling was undertaken only where two or more trials reported an outcome with a commensurable operational definition, denominator, and effect metric; this criterion, rather than study quality, determined which trials contributed to meta-analysis, and yielded two poolable outcome families (appropriate renal dosing and EHR-documented CKD recognition). Trials reporting related but non-equivalent outcomes (e.g., continuous SBP change versus dichotomous BP control) were retained in the narrative and structured syntheses but not pooled; per-trial reasons are given in Table 8.

**Table 8.** Randomized controlled trials included in the review but not entered into meta-analysis, and the reason each was non-commensurable with a poolable outcome.

Trial (year) [ref]	Outcome(s) reported	Effect as reported	Reason not commensurable with a poolable set
Abdel-Kader (2011) ^48^	Nephrology referral; albuminuria/ACR testing	9.7% vs 16.5% (P = 0.10); 39.3% vs 30.1% (P = 0.10)	Referral/testing outcomes, not the CKD-diagnosis documentation of the pooled recognition pair; no commensurable partner.
Alhodaib (2020) ^37^	Clinician decision-accuracy score; task time	7.24 vs 7.39 (P=0.85)	Clinician-level score from simulated case scenarios; unique construct.
Carroll (2018) ^42^	CKD diagnosis on problem list; NSAID avoidance; ACEi/ARB use	Process outcomes “no significant difference”; effect sizes not reported	Overlaps the recognition construct but reports no extractable effect size.
Chen (2022) ^39^	NRS-2002 nutritional risk; anthropometry; SF-36	NRS-2002 improved in H2H arm (P<0.05)	Nutrition/quality-of-life domain, outside the pooled safety constructs.
Erler (2012) ^35^	Patients with ≥1 prescription exceeding maximum daily dose	OR 0.46 (95% CI 0.26–0.82); 19.2% vs 34.5%	Patient-level “any overdose” (OR), not order-level appropriateness (RR).
Januzzi (2025) ^40^	Cardio-kidney risk prediction (composite)	Model performance only; no CDSS deployed	Prediction-model development only; no comparative CDSS process outcome.
Lim (2025) ^36^	Mean Hb deviation from 11 g/dL; Hb 10–12 g/dL; deaths	Hb 10–12 g/dL 69.7% vs 73.5%; deaths 1 vs 1	ESA/anemia control; model-selection design; per-test denominators not extractable.
Locatelli (2009) ^45^	Hb >11 g/dL; ferritin >100 ng/mL	Hb >11 g/dL 67% vs 70% (P NR)	Dialysis-anemia target attainment; unique domain; denominators not reported.
Mancini (2007) ^38^	Severe intradialytic hypotension (% sessions)	8.3% vs 13.8% (P=0.01)	Session-level crossover design, incompatible with parallel-group pooling.
Patzer (2018) ^46^	Transplant knowledge score; access composite	Knowledge difference-in-differences 0.71 (P<0.001)	Transplant-education domain, outside the pooled safety constructs.
Peralta (2020a) ^44^	New RAS-inhibitor use; CKD recognition among newly-detected	RAS new use 7% vs 10% vs 10% (P=0.23)	Recognition denominator and RAS new-use definition differ from poolable outcomes.
Samal (2022) ^43^	KFRE-lab completion; nephrology referral (high-risk)	Labs 6.95% vs 6.56% (P=0.61)	Monitoring/referral construct; denominator derivation not fully specified.
Samal (2024) ^41^	Action aligned with CDS; SBP change; any ACE/ARB/HCTZ order	Action 49.9% vs 34.6% (P<0.001); SBP −14.6 vs −11.7 mmHg (P=0.005)	Continuous SBP change (mean difference) vs dichotomous BP control; differing ACEi/ARB denominator.
Sequist (2018) ^47^	Nephrology visit (high-risk); microalbumin testing (low-risk)	Nephrology visit 45% vs 34% (P<0.001)	Risk-stratified referral/testing; different construct and denominator.

Only two outcome families were reported by two or more trials with a commensurable operational definition, denominator, and effect metric (appropriate renal dosing, four trials; EHR-documented CKD recognition, two trials); the 14 trials above could not be combined with any poolable set. Effect sizes are reproduced as reported. Abbreviations: ACEi, angiotensin-converting enzyme inhibitor; ACR, albumin-to-creatinine ratio; ARB, angiotensin receptor blocker; BP, blood pressure; CDS(S), clinical decision support (system); CKD, chronic kidney disease; ESA, erythropoiesis-stimulating agent; Hb, hemoglobin; HCTZ, hydrochlorothiazide; H2H, hospital-to-home; KFRE, kidney failure risk equation; NR, not reported; NRS-2002, Nutritional Risk Screening 2002; OR, odds ratio; RAS, renin–angiotensin system; RR, risk ratio; SBP, systolic blood pressure.

### 2.7 Certainty of evidence

We did not apply GRADE because outcomes and effect measures were highly heterogeneous and many trials did not report estimable effect sizes suitable for consistent certainty rating across outcomes.

## 3. Results

### 3.1 Study selection

**Figure 1.**
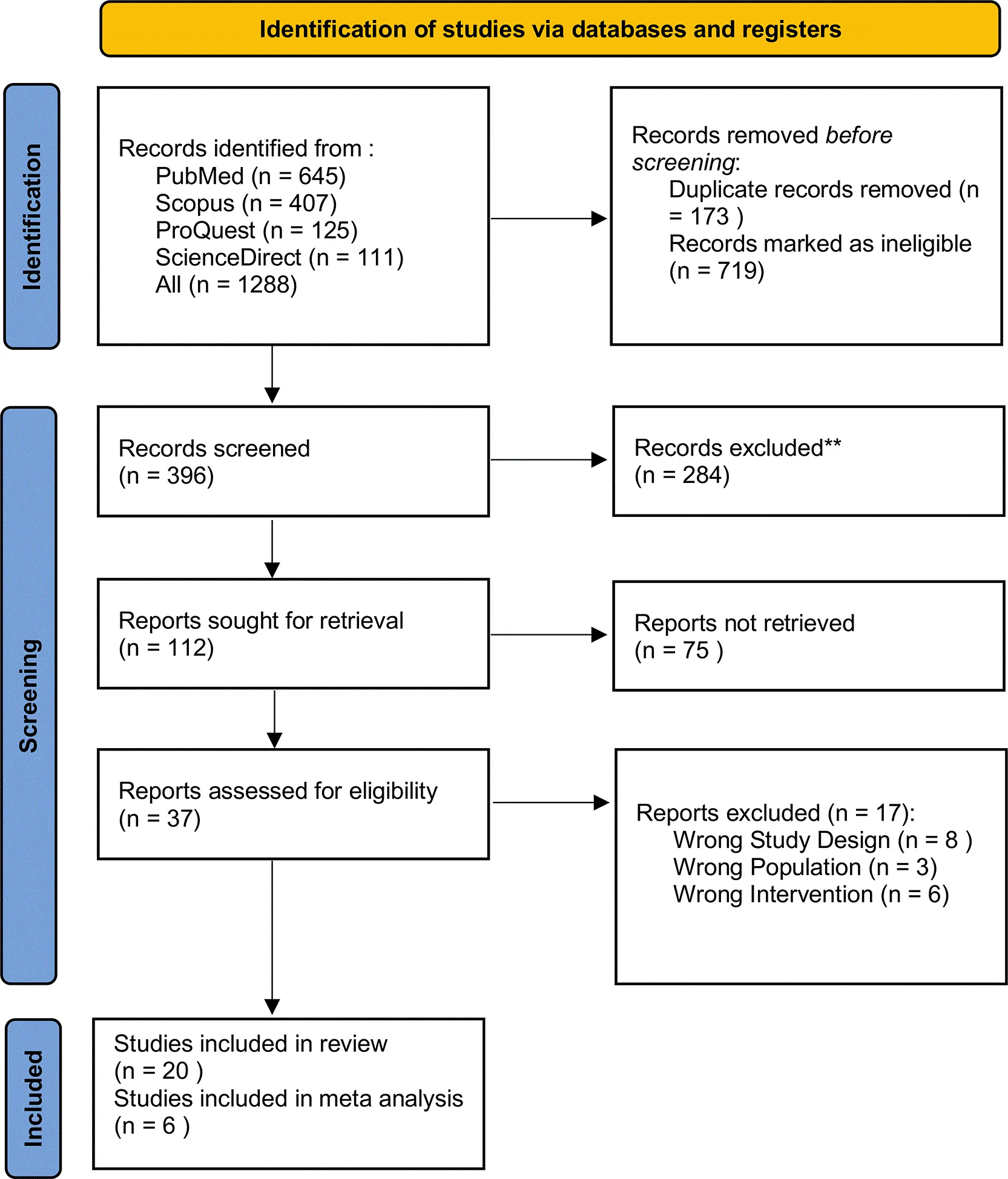
PRISMA flow diagram of study identification, screening, eligibility assessment, and inclusion for randomized trials of CDSS in chronic kidney disease.

Based on the developed search strategy, 1,288 articles were identified through systematic searching. Of these, after removing 173 duplicates, 396 articles were screened by title and abstract. Then, for reasons not meeting the inclusion criteria, 284 were excluded. We sought retrieval of 112 reports, of which 75 were not retrieved, leaving 37 reports assessed for eligibility. In total, 20 trials met our inclusion criteria, and 6 of them underwent quantitative analysis. The two poolable outcome families and the specific reason each of the remaining 14 trials could not contribute to pooling are documented in Table 8. Figure 1 depicts the process of literature retrieval.

### 3.2 Study characteristics

The main characteristics of the included studies are summarized in Table 3. The CDSS interventions we examined were heterogeneous in terms of their technical platform (eg., combined EHR/CPOE systems to standalone applications for mobile platforms) and core functions (eg., renal dosing support or CKD documentation). To consider this diversity as the first major contributory source of heterogeneity, we sought to control for it upfront using an organized Best Evidence Synthesis (BES).

**Table 3.** Characteristics of the included studies.

Source (First author, Publication year)	Year	Country	Trial designs	Number of participants	Specific technology/platform used
Abdel-Kader, 2012 ^48^	2011	United States	Cluster randomized controlled trial	248	EpicCare (Epic Systems)
Alhodaib, 2020 ^37^	2020	United Kingdom	Randomized controlled trial (RCT)	54 (junior doctor and specialist nurses)	Mobile app developed by Medic Genie
Awdishu, 2016 ^29^	2016	United States	Cluster randomized controlled trial	1278	Epic EHR
Bhardwaja, 2011 ^32^	2011	United States	Randomized controlled trial (RCT)	32917	Drug Renal Alert Pharmacy (DRAP) program
Carroll, 2018 ^42^	2018	United States	Cluster randomized controlled trial	6699	Three separate systems were used due to vendor issues; integrated with electronic health records (EHRs).
Chen, 2022 ^39^	2022	China	Randomized controlled trial (RCT)	120	Internet + H2H model
Erler, 2012 ^35^	2012	Germany	Cluster randomized controlled trial	404	DOSING software (standalone CD version)
Field, 2009 ^30^	2009	Canada	Cluster randomized controlled trial	800 (residents)	Meditech MAGIC platform using Provider Order Management (POM4.9 upgraded to 5.5)
Januzzi, 2025 ^40^	2025	The study was conducted in 34 countries with significant European representation.	Randomized controlled trial (RCT)	4401	Machine learning-based risk prediction algorithm for diabetic kidney disease (DKD).
Lim, 2025 ^36^	2025	Taiwan	Randomized controlled trial (RCT)	124	The study involved four machine learning models: bagged Regression trees with random effects (REEM) trees, Mixed-effect random forest (MERF), Long short-term memory (LSTM) networks LSTM-I, and LSTM-II.
Locatelli, 2009 ^45^	2009	Bulgaria, Croatia, Germany, Italy, Latvia, Poland, Romania, Serbia and Montenegro	Cluster randomized controlled trial	599	Interactive centrally controlled database with secure Internet access case report form and a CDS system generating guideline-based management prompts.
Mancini, 2007 ^38^	2007	Italy	Crossover RCT	55	Dialog Advanced dialysis machine by Braun, integrated with bioLogic RR system.
Patzer, 2018 ^46^	2018	United States	Randomized controlled trial (RCT)	443	Mobile and web-based application
Peralta, 2020 ^44^	2020	United States	Cluster randomized controlled trial	1819	Electronic Health Record (EHR)
Peralta 2020 ^34^	2020	United States	Cluster randomized controlled trial	524	EpicCare electronic health record (EHR) system
Samal, 2022 ^43^	2022	United States	Randomized controlled trial (RCT)	5590	Web-based application with a single-page interface, web server, database, and application programming interface.
Samal, 2024 ^41^	2024	United States	Cluster randomized controlled trial	2026	Epic Systems software
Sequist, 2018 ^47^	2018	United States	Cluster randomized controlled trial	7691	Epic Systems EHR
Sperl-Hillen, 2023 ^33^	2023	United States	Cluster randomized controlled trial	6420	EpiCare electronic health record (EHR) system
Terrell 2010 ^31^	2010	United States	Randomized controlled trial (RCT)	2783	The AI-CDSS intervention is a computerized decision support system integrated into a computerized physician order entry (CPOE) system.

Abbreviations: CDSS = Clinical Decision Support System; AI = Artificial Intelligence; EHR = Electronic Health Record; CPOE = Computerized Provider Order Entry; RCT = Randomized Controlled Trial. Specific technology/platform includes EHR/CPOE-integrated systems, web-based platforms, mobile applications, and standalone software; EHR embedding was not required for eligibility.

To provide a view beyond common pooled estimates, we used a layered model for evidence synthesis. This comprised an Evidence Map ( Figure 4) to provide an overview of coverage across outcome domains, and a Best-Evidence Table ( Table 7) identifying robust patterns and areas of poor reporting within each configuration. Finally, we illustrated the ‘architecture’ of this evidence with a Network Map ( Figure 5) that outlines how details from particular trials and implementation configurations convert into outcome domains.

### 3.3 Risk of bias in included studies

**Figure 2.**
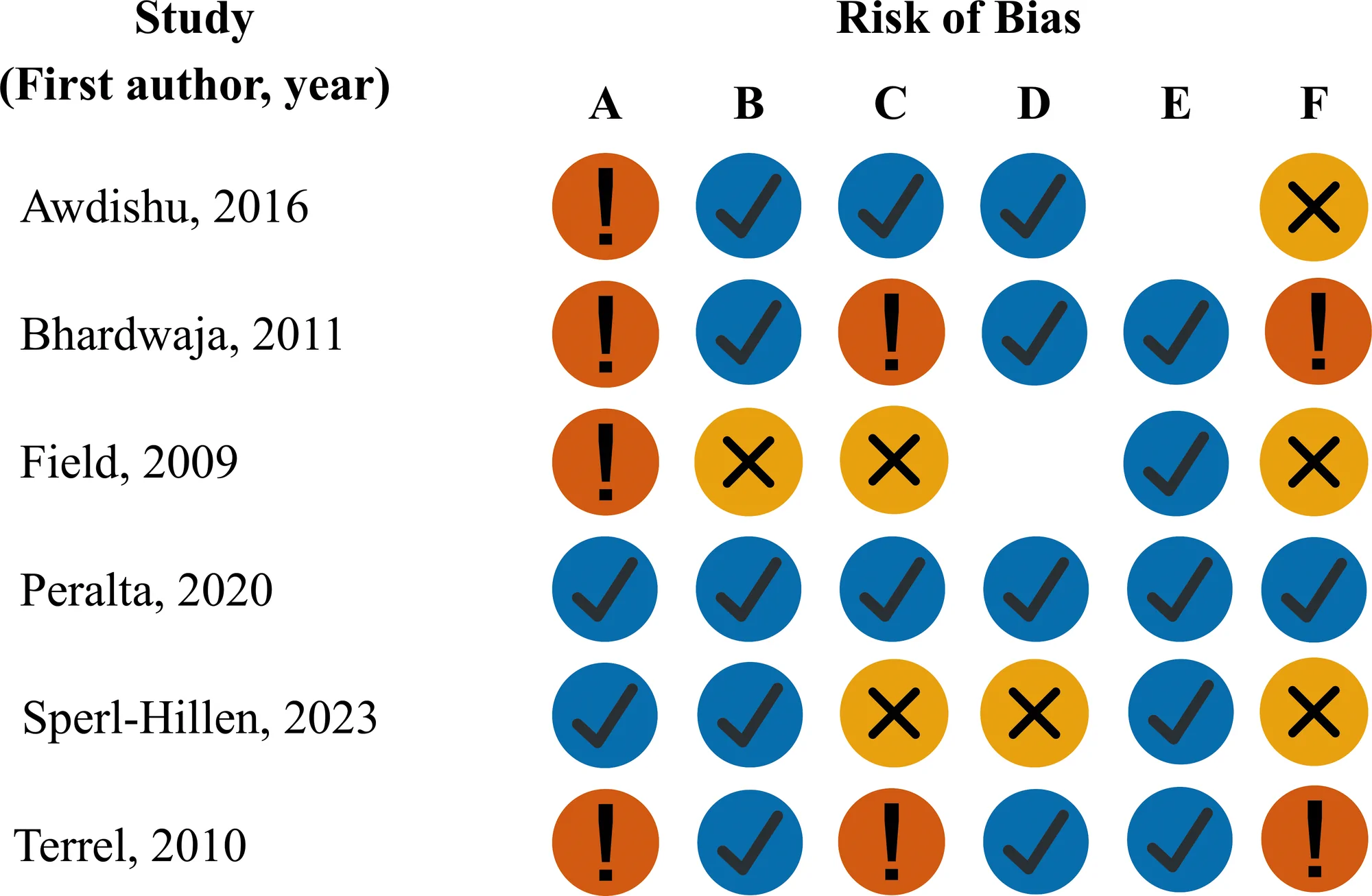
RoB 2 risk-of-bias assessment for the randomized controlled trials included in this review. Each row represents a study and columns A–F represent domains (A, randomization process; B, deviations from intended interventions; C, missing outcome data; D, measurement of the outcome; E, selection of the reported result; F, overall bias), with blue = low risk (√), orange = some concerns (!), and vermilion = high risk (X).

For the six studies included in the meta-analysis, the risk-of-bias assessment is shown in Figure 2. For the first outcome, 4 RCTs were generally at high risk or unclear in several domains (e.g. deviations from intended intervention, missing outcome data, selection of the reported result); underestimation of the outcome was largely low risk ^29– 32^; this corresponds with very high heterogeneity observed (I ^2^ = 97%; pooled RR 1.76, 95% CI 1.13–2.74). For the second outcome, Peralta (2020) was low risk across all domains, whereas Sperl-Hillen (2024) identified several domains as high risk. ^33, 34^ Effect consistency in EHR-documented CKD banner was high (I ^2^ = 0%) with precise estimates (fixed-effect: RR 1.20 [1.08–1.33]; random-effects: RR 1.19 [1.07–1.32]). Risk-of-bias diagrams are shown below ( Figure 2).

### 3.4 Primary outcome

**3.4.1 Appropriate renal dosing**

CDSS significantly improved appropriate renal dosing compared with standard care, with a relative risk (RR) of 1.76 (95% CI 1.13–2.74; Z = 2.51; p = .01). But there was considerable heterogeneity among the studies included, and I ^2^ = 97%, τ ^2^ = 0.19, χ ^2^ = 72.66, df = 3, p < 0.00001. Therefore, a random-effects model was used to pool results across four RCTs. ^29– 32^

**Figure 3.**
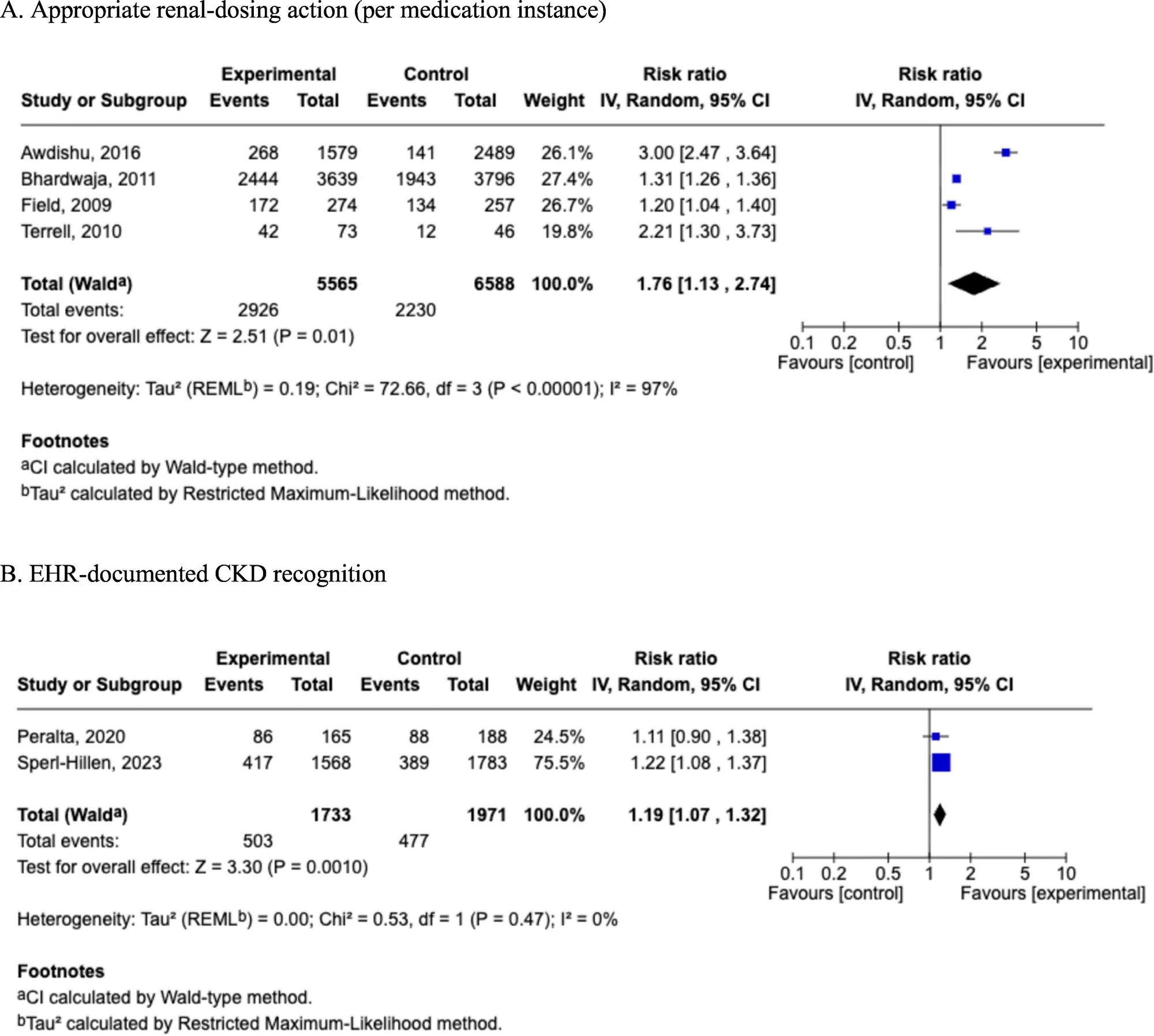
Forest plots of the effect of CDSS versus usual care on appropriate renal-dosing actions per medication instance (A) and on EHR-documented CKD recognition (B); squares represent study-specific risk ratios with 95% confidence intervals weighted by inverse variance, diamonds indicate the pooled random-effects estimates, and the vertical line at RR = 1.0 denotes no difference between groups. A. Appropriate renal-dosing action (per medication instance). B. EHR-documented CKD recognition.

The effect of these interventions was highly varied; study-specific effects ranged from RR 1.20 to 3.00. This wide range illustrates how success is highly influenced by the clinical context and implementation approach. In absolute numbers, the CDSS arms reached appropriate dosage in 2,926 out of 5,565 medication instances-significantly more frequent than the same figure in control groups (2,230/6,588) ( Figure 3).

**3.4.2 Prescribing and medication safety**

Most studies show that an advantage works for rule-driven CDSS. In seven studies assessing for the dosage/prescription of medication, five were in favor of CDSS, including reductions in overdose (19.2% vs 34.5%, OR 0.46; 95% CI 0.26–0.82; p = 0.008), ^35^ reductions in excessive dosage (43% vs 74%, p = 0.001), ^31^ fewer medication errors (33% vs 49%, p < 0.001), ^32^ various measures for proper dosage across many subcomponents (RR doses 0.95; Frequency 2.4; Avoidance 2.6; Information 1.8), ^30^ and higher rates for suitable renal dosage (17% vs 5.7%, OR 1.89; 95% CI 1.45–2.47; p < 0.001). ^29^ One study found no significant difference in prescription failure (1.26% vs 0.5%, p = 0.11), ^36^ and another was neutral regarding workflow/guideline outcomes. ^37^ Beyond dosage/prescription, one hemodialysis study reported reduced severe hypotension (8.3% vs 13.8%; p = 0.01), ^38^ and one study of nutritional/biochemical changes showed improvements in parameters (albumin, prealbumin, hemoglobin, BUN), anthropometrics, quality of life, and satisfaction. ^39^ One risk prediction study did not apply clinical implementation, ^40^ while the other provided no outcome data or direction of effect. Overall, evidence in the domain of medication safety and appropriateness supports CDSS; however, effect sizes and outcome types differ across studies.

**3.4.3 Best Evidence Synthesis (BES) by implementation configuration**

Magnitude of effect was diverse, with study-specific RRs ranging from 1.20 to 3.00. While the summary of raw values points towards a benefit in the CDSS arms (2,926 doses correct out of 5,565 as compared to 2,230/6,588 in controls), heterogeneity is marked (I ^2^ = 97%) and any one average effect is virtually meaningless. This initiates our attention for a Best Evidence Synthesis, to investigate why certain settings work better than others.

**Figure 4.**
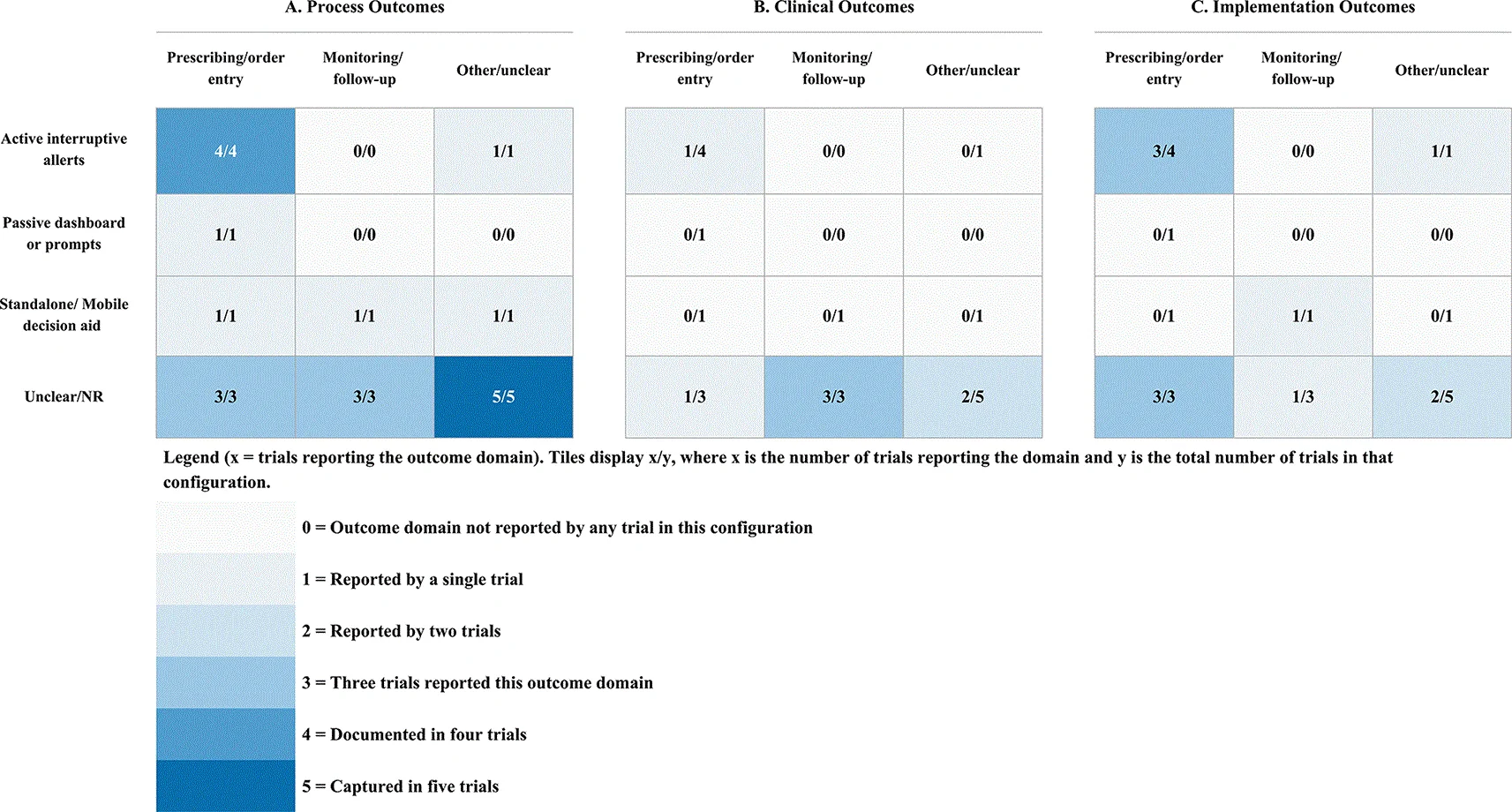
CDSS implementation settings (Best evidence synthesis; Evidence map of outcome reporting). This map illustrates how reporting is distributed across three dimensions: (A) process, (B) clinical, and (C) implementation outcomes. The modes of delivery (vertical axis; e.g., active, interruptive alerts vs. passive dashboards) and timing in the workflow (horizontal axis; e.g., prescribing to avoid monitoring) can be grouped under this framework. Values within each tile indicate the percentage of trials (in that category) reporting a particular outcome. The density of evidence, on a scale of 0–5, is depicted as a color scale, with darker colors indicating greater accumulated evidence. The map reveals that, while process results are well reported, clinical and implementation outcomes further downstream are inconsistently collected. This discrepancy highlights a significant need for core outcome and KPI reporting to be standardised in future CDSS trials.

**Figure 5.**
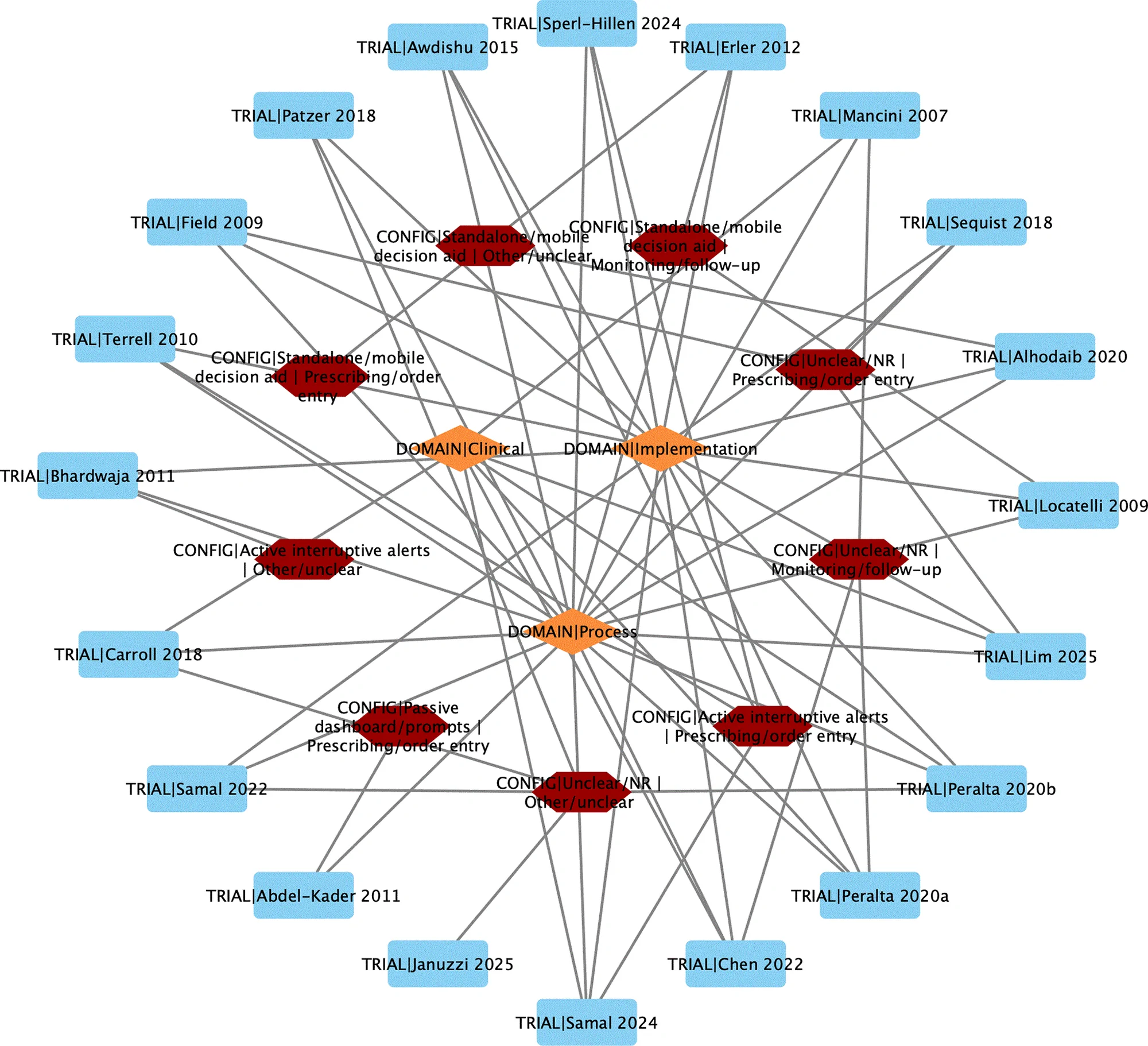
Design of CKD CDSS evaluation trial architecture at the level of evidence: central configuration and domain connections. Network showing how each randomized and quasi-randomized study was linked to implementation strategies (delivery mode × workflow timing) and the outcome domains reported. Tested configurations nodes (blue) are connected to a single configuration node (red) representing the intervention’s delivery–timing and domain nodes (orange), reflecting whether the trial reported process, clinical, or implementation outcomes. This trial-based network architecture underscores that the evidence weight, in terms of structural debris, is aggregated at a relatively few configuration hubs, with links to central (clinical) nodes relying on a subset of trials—suggesting potential (evidence) over-biasedness and fragility with respect to clinical impacts.

Our mapping of trials by delivery method and workflow timing reveals a lopsided evidence base. While there is no shortage of data on how interruptive alerts affect prescribing errors, the evidence regarding long-term clinical significance and implementation hurdles remains thin and varies wildly between settings (see Figure 4). This gap between process-level success and clinical impact is critical. We’ve summarized the specific signals and reporting deficiencies for each configuration in Table 7. Finally, Figure 5 gives a network-level perspective on the ‘architecture’ of the evidence base; we can see which trials support each configuration, and in what area of the evidence base fragility remains. A more detailed trial-by-trial mapping can be found in the Extended data (Appendix 2).

### 3.5 Secondary outcomes

**3.5.1 EHR-documented CKD recognition**

CDSS performed significantly better than standard care in identifying documented CKD in the EHRs (relative risk [RR] = 1.19, 95% confidence interval [CI] = 1.07 to 1.32; Z = 3.30, p = 0.001). There was no heterogeneity (I ^2^ = 0%; τ ^2^ = 0.00; χ ^2^ = 0.53; df = 1; P = 0.47). Pooled data from two RCTs were analyzed using a random-effects model based on weighted inverse variance with restricted maximum likelihood (REML) estimation. The study-specific estimates were Peralta (2020) ^34^ (RR = 1.11 [0.90–1.38]; weight, 24.5% [86/165 vs 88/188]) and Sperl-Hillen (2024) ^33^ (RR = 1.22 [1.08–1.38]; weight, 75.5% [417/1568 vs 389/1783]). A total of 503 of 1733 events occurred in the CDSS arms vs. 477 of 1971 in the control arms. A summary of aggregated effects under fixed- and random-effects models is presented in Table 4.

**Table 4.** Summary of meta-analytic results (fixed-effect vs. random-effects).

Outcome	Method	Effect measure	Pooled effect (95% CI)	Heterogeneity (Q; df; p; I ^2^)	τ ^2^ (Estimator)	95% Prediction interval	Notes
Appropriate renal dosing	M-H, Fixed	RR	1.40 (1.35–1.45)	72.66; 3; <0.00001; 96%	—	—	FE (fixed-effect) is shown as a comparator; assumes a single “true effect.”
Appropriate renal dosing	IV, Random	RR	1.76 (1.13–2.74)	72.66; 3; <0.00001; 97%	0.19 (REML)	0.75–4.14	Primary model; weights are more balanced across studies; confidence intervals are wider due to high heterogeneity.
EHR-documented CKD recognition	M-H, Fixed	RR	1.19 (1.07–1.32)	I ^2^ = 0%	—	—	Zero heterogeneity; FE ≈ RE (random-effects).
EHR-documented CKD recognition	IV, Random	RR	1.19 (1.07–1.32)	I ^2^ = 0%	≈0.00 (REML)	[≈ same]	Identical to FE when I ^2^ = 0%.

Abbreviations: M-H = Mantel–Haenszel method; IV = Inverse Variance method; RR = Risk Ratio; CI = Confidence Interval; Q = Cochran’s Q; df = degrees of freedom; I ^2^ = Inconsistency index; τ ^2^ = Between-study variance; REML = Restricted Maximum Likelihood; FE = Fixed-effect; RE = Random-effects; CKD = Chronic Kidney Disease; EHR = Electronic Health Record.

**3.5.2 Mortality events**

Two studies provided mortality data. In the single randomized hemodialysis trial (Lim 2025), ^36^ there was no difference in mortality between AI-CDSS and conventional care (one death in each arm). Other significant adverse events, such as hospital admission and blood transfusion, also showed similar results (7 versus 9 and 5 versus 5). ^36^ Second, the other study did not assess the impact of the CDSS intervention in clinical practice but rather aimed to develop and validate a machine-learning-based risk algorithm. Mortality (renal and cardiovascular) was a non–treatment-related available future risk predictor, but there was no clinical practice evaluation of the CDSS, ^40^ Consequently, it can be concluded that we were unable to assess intervention effect on mortality, despite its inclusion in that trial among the predicted composite outcomes assessed from treatment or control status; however, as alluded to earlier this means that study had to be excluded from the meta-analysis for subject outcome specifications on Mortality in Table 5.

**Table 5.** Mortality and major clinical events.

Study	Outcome category	AI-CDSS results	Comparator results	Effect direction
Lim, 2025 ^36^	Death events	No difference	Standard care	Neutral
Januzzi, 2025 ^40^	Cardiovascular/renal death	Model predicts, not tested in practice	Not applicable	Not applicable

Abbreviations: AI-CDSS = Artificial Intelligence–based Clinical Decision Support System. Effect direction: “Neutral” = no statistically significant difference between AI-CDSS and comparator. “Not applicable” = mortality/major clinical events were not tested in a comparative trial (model development only).

**3.5.3 Renal function**

The evidence is very suggestive of a beneficial effect of the intervention groups on general renal function and certain specific physiological outcomes. However, the findings show varied results across different types of outcomes. The benefits of the CDSS intervention were described in two studies. One trial that used a rule-based CDSS reported the estimated Glomerular Filtration Rate slope to decline less steeply when facilitation was added (−0.01 with CDSS plus facilitation vs − 0.95 with CDSS alone; p < 0.001; favors intervention), ^42^ Another AI-CDSS study also showed an increase in renal cortical blood flow (p < 0.05; between groups). ^39^ Three other studies were of neutral or no difference around hemoglobin control (within target with AI-CDSS versus physician management), ^36^ CKD quality metrics, ^33^ and laboratory monitoring. ^43^ One study was for risk prediction model development, not clinical implementation, therefore presenting no comparative effect data; and the other did not report the data or direction for the outcome. ^40^

**3.5.4 Blood Pressure (BP) control and Cardiovascular (CV) outcomes**

The evidence for the potential utility of Clinical Decision Support Systems (CDSS) across a range of CV and BP-related outcomes is cumulative. At the same time, results vary across BP measures in the included studies—mixed directionality of effect. Five studies showed mixed effects: three favored the CDSS and two were neutral. Regarding blood pressure-related outcomes (change or control; three studies), one showed a decrease in systolic blood pressure (SBP) that was significantly greater among the CDSS arm as compared to the standard care group (-14.6 mmHg for CDSS vs -11.7 mmHg for control; p = 0.005), ^41^ whereas two others did not find differences in terms of either blood pressure control ^42^ or change. ^44^ Furthermore, a single study reported improved quality of life (favoring AI-CDSS), ^39^ and another study reported a decreased rate of severe intradialytic hypotension (also favoring the AI-CDSS). ^38^ Quantitative effect sizes and p-values were reported exclusively in one study; the other four reported only the direction of effect without providing numerical values.

**3.5.5 Usability and provider acceptance**

Clinicians’ involvement in CDSS was highly heterogeneous in this context. Provider-level adherence in five studies with quantitative data ranged from 17% to 74%. ^29, 31, 34, 45^ Only two studies included qualitative terms to define engagement (low use and high compliance) ^43, 46^ while four studies reported no adherence outcomes. ^32, 33, 36, 37^ User satisfaction was quantitatively evaluated in two studies and showed low or no burden, with high satisfaction rates of 74% and 82% among users, ^37, 44^ respectively. Barriers for (non-)implementation and use were reported in eight studies, among others, alert fatigue, lack of training, time pressures, unfamiliarity with CDSs or resistance to change, technical problems like application crashes, disabling printing, economic reasons, disturbances relating to COVID-19, and overall burden of users on time. ^29, 31, 33, 36, 37, 43, 45, 46^ Regarding the facilitators, they were cited in two studies that reported integration of their results and a reduction in false positives. ^32, 34^ Sustainability was reported in three papers: two reported plans to continue or increase use, and one found sustained error reduction. ^31, 32, 46^ In general, clinician acceptance of CDSS was moderate and varied considerably; there is potential to further support implementation by integrating systems to improve CDSS adoption.

**3.5.6 Guideline adherence and clinical process measures**

For guideline adherence and process measures, two studies favored AI-CDSS implementation, two were neutral, and one reported no effect direction. AI-CDSS was linked to increased ACEi/ARB utilization and nephrology referral, and decreased medication errors. ^32, 47^ In contrast, adherence to ESA was not inferior to usual care or low, which probably reflects human/systemic barriers. ^36, 45^ Heterogeneity of outcomes prevented pooling; overall, benefits seem to be aimed at prescribing/referral and medication security, with adherence possibly requiring more than decision support ( Table 6).

**Table 6.** Guideline adherence and clinical process measures.

Study	Outcome	AI-CDSS results	Comparator	Effect direction
Lim, 2025 ^36^	ESA guideline adherence	Non-inferior	Standard care	Neutral
Sequist, 2018 ^47^	ACEi/ARB, nephrology referral	Improved in high-risk	Standard care	Favors CDSS
Bhardwaja, 2011 ^32^	Medication errors	Reduced	Standard care	Favors CDSS
Locatelli, 2009 ^45^	Adherence	Low even with CDSS	Standard care	Neutral

Abbreviations: ESA = Erythropoiesis-Stimulating Agent; ACEi = Angiotensin-Converting Enzyme inhibitor; ARB = Angiotensin Receptor Blocker; CDSS = Clinical Decision Support System; AI-CDSS = Artificial Intelligence–based Clinical Decision Support System. Effect direction: “Favors CDSS” = AI-CDSS shows statistically or clinically meaningful improvement vs standard care; “Neutral” = No clear difference between AI-CDSS and comparator.

## 4. Discussion

This systematic review of 20 RCTs demonstrates that renal-CDSS principally enhance proximal medication safety; however, their impact on downstream clinical outcomes is unclear. ^29– 48^ Most interventions were guideline-based rule systems incorporated into electronic prescribing or EHRs, with a minority being AI-powered tools; compared with control conditions across over 74,000 patients and 854 health professionals, CDSS uniformly changed prescribing behavior towards safety (not clearly reducing mortality or progression to kidney failure). Because the implementation of the trials was so varied, in each case, we felt a pooled estimate would disguise as much as it would reveal. We thus supplemented our quantitative data with a structure-based synthesis and granular, trial-by-trial mapping ( Figures 4 and 5, Table 7, and Table S2). This framing takes the discussion beyond a dichotomous question of whether CDSS ‘works’. It reveals the real underlying structure of the research: where exactly the evidence lies in relation to different delivery modes and workflow stages — and, crucially, where, down the line, clinician evaluations are missing.

**Table 7.** Configuration-dependent signals and minimum reporting needs for CKD CDSS trials (Best evidence synthesis).

Delivery mode	Workflow timing	Trials (n)	Included trials (first author, year)	Dominant function	Primary process target(s) (examples)	Process signal (trial-level)	Clinical endpoints evaluated (availability; summary)	Implementation measurement (availability; key types)	Recurrent missing elements (to enable future pooling)
Active interruptive alerts	Prescribing/order entry	4	Awdishu 2015 ^29^; Samal 2024 ^41^; Sperl-Hillen 2024 ^33^; Terrell 2010 ^31^	Dosing	Renal dosing/appropriate prescribing	Favors 3/4; Neutral 1/4	1/4; mixed	4/4; adoption/use, adherence, alert burden, overrides	governance/update; severity tiering; ownership
Active interruptive alerts	Other/unclear	1	Bhardwaja 2011 ^32^	Dosing	Medication errors; false positives	Favors 1/1	0/1; not evaluated	1/1; adoption/use, adherence, burden/satisfaction	severity tiering; override reasons; governance detail
Passive dashboard/prompts	Prescribing/order entry	1	Abdel-Kader 2011 ^48^	Recognition	Referral/testing prompts	Mixed 1/1	0/1; not evaluated	0/1; NR	implementation descriptors variably NR (see Table S1)
Standalone/mobile decision aid	Prescribing/order entry	1	Erler 2012 ^35^	Dosing	Renal dosing exceedance	Favors 1/1	0/1; not evaluated	0/1; NR	alert modality; trigger logic; denominators
Standalone/mobile decision aid	Monitoring/follow-up	1	Locatelli 2009 ^45^	Monitoring	Hb/ferritin thresholds	Neutral 1/1	0/1; not evaluated	1/1; adoption/use, alert burden, satisfaction	alert modality; trigger logic; rule transparency
Standalone/mobile decision aid	Other/unclear	1	Alhodaib 2020 ^37^	Monitoring	Decision accuracy score	Neutral 1/1	0/1; not evaluated	1/1; adoption/use, burden/satisfaction	workflow efficiency NR; modality/triggering; integration detail
Unclear/NR	Prescribing/order entry	3	Field 2009 ^30^; Lim 2025 ^36^; Sequist 2018 ^47^	Dosing/Monitoring	Appropriate orders/targets	Favors 1/3; Mixed 2/3	1/3; no difference	3/3; adoption/use, burden, overrides	modality classification; severity tiering; interface/trigger detail
Unclear/NR	Monitoring/follow-up	3	Chen 2022 ^39^; Mancini 2007 ^38^; Peralta 2020 ^44^	Monitoring/Recognition	Monitoring risks (e.g., hypotension/nutrition/CKD recognition)	Favors 3/3	3/3; mixed	2/3; adoption/use, satisfaction	reporting gaps vary (see Table S1)
Unclear/NR	Other/unclear	5	Carroll 2018 ^42^; Januzzi 2025 ^40^; Patzer 2018 ^46^; Peralta 2020 ^34^; Samal 2022 ^43^	Recognition/Risk prediction	CKD recognition/awareness; risk-tool labs	Favors 1/5; Neutral 1/5; Mixed/Worse/NR remaining	3/5; mixed/NR/no difference	3/5; adoption/use, burden/satisfaction	modality; trigger logic; required inputs

Abbreviations: CKD = Chronic Kidney Disease; CDSS = Clinical Decision Support System; NR = Not Reported. Configuration taxonomy: delivery mode refers to how decision support is delivered (active interruptive alerts, passive dashboards/prompts, standalone/mobile decision aid, or unclear/NR); workflow timing refers to when decision support is applied (prescribing/order entry, monitoring/follow-up, or other/unclear). Effect direction (process signal): “Favors CDSS” = trial-level process outcomes show a statistically significant or clinically meaningful improvement in the CDSS arm vs comparator; “Neutral” = no clear difference between arms; “Mixed” = outcomes or subcomponents show inconsistent direction within the trial; “Worse” = process outcomes favor the comparator; “NR” = direction not reported or cannot be determined from the trial report. Domain availability: “Clinical endpoints evaluated” and “Implementation measurement” indicate whether these domains were assessed and reported for that configuration (see Figure 4 for overall reporting distribution).

Of the 20 included trials, only six could be pooled, because meta-analysis requires at least two trials reporting an outcome with a commensurable operational definition, denominator, and effect metric. The other 14 trials measured related but non-equivalent constructs. Table 8 sets out, trial by trial, the specific reason each could not be entered into meta-analysis.

The eligible trials also varied in the renal population they enrolled — from threshold-defined renal impairment (creatinine-clearance or eGFR cut-offs) through staged CKD to prevalent dialysis. Table 9 characterizes each included trial by its target population and indicates which trials contributed to each meta-analysis.

**Table 9.** Target renal population of each included trial.

Trial (year) [ref]	Population as enrolled	Renal stratum	In meta-analysis?
Abdel-Kader (2011) ^48^	eGFR <45, non-dialysis	CKD ≈G3b–G4	—
Alhodaib (2020) ^37^	Type 2 DM + CKD; stage NR	Diabetic CKD (stage NR)	—
Awdishu (2016) ^29^	Adults >18, CLcr <60; dialysis excluded	Threshold-defined renal impairment	Pooled — appropriate dosing
Bhardwaja (2011) ^32^	Adults ≥18, CrCl ≤50; dialysis excluded	Threshold-defined renal impairment	Pooled — appropriate dosing
Carroll (2018) ^42^	CKD stage 3–4 (eGFR 15–60)	CKD G3–G4	—
Chen (2022) ^39^	CKD stage 3–5, stable; RRT excluded	CKD G3–G5	—
Erler (2012) ^35^	eGFR <50 + CKD codes, or ≥70 with HTN	Renal impairment/at-risk elderly	—
Field (2009) ^30^	Renal insufficiency (~81% CrCl <60)	Threshold-defined renal impairment	Pooled — appropriate dosing
Januzzi (2025) ^40^	DKD (eGFR 30–90, UACR >300)	Diabetic kidney disease (prediction cohort)	—
Lim (2025) ^36^	ESKD on maintenance HD	Dialysis	—
Locatelli (2009) ^45^	CKD G5 on dialysis, renal anemia	Dialysis (anemia)	—
Mancini (2007) ^38^	Maintenance HD, hypotension-prone	Dialysis	—
Patzer (2018) ^46^	ESRD transplant candidates (~⅓ pre-dialysis)	Advanced CKD/ESKD	—
Peralta (2020a) ^44^	Nondiabetic HTN veterans; no CKD at baseline	At-risk/screening	—
Peralta (2020b) ^34^	eGFR 30–59 (CKD-EPI) ×2, ≥90 d apart	CKD G3	Pooled — CKD recognition
Samal (2022) ^43^	CKD stage 3–5, eGFR <59	CKD G3–G5	—
Samal (2024) ^41^	CKD stage 3–4 (eGFR 16–59) with HTN	CKD G3–G4 + hypertension	—
Sequist (2018) ^47^	Stage III CKD, eGFR 30–60	CKD G3	—
Sperl-Hillen (2024) ^33^	Laboratory-defined CKD G3–G4	CKD G3–G4	Pooled — CKD recognition
Terrell (2010) ^31^	Adults ≥18, CrCl below drug-specific threshold	Threshold-defined renal impairment	Pooled — appropriate dosing

The four pooled appropriate-dosing trials defined their population by a creatinine-clearance/eGFR threshold (mixed renal impairment) rather than by CKD stage; the two pooled recognition trials enrolled staged CKD G3–G4 primary-care populations; a distinct dialysis cluster was not pooled. Peralta (2020a) ^44^ (CKD screening) and Peralta (2020b) ^34^ (CKD management) are distinct trials; only the latter contributed to the recognition meta-analysis. Abbreviations: CKD-EPI, Chronic Kidney Disease Epidemiology Collaboration; CLcr/CrCl, creatinine clearance; DKD, diabetic kidney disease; DM, diabetes mellitus; eGFR, estimated glomerular filtration rate; ESKD/ESRD, end-stage kidney/renal disease; HD, hemodialysis; HTN, hypertension; NR, not reported; RRT, renal replacement therapy; UACR, urine albumin-to-creatinine ratio.

### 4.1 Proximal medication-safety gains are consistent and clinically meaningful

The most consistent benefit to patient safety is better renal-dose appropriateness and its prescribing. Studies that included real-time alerts or order-entry checks resulted in higher doses based on kidney function, decreased use of contraindicated drugs, and reduced excessive dosing of renally eliminated drugs. ^29– 32, 35, 38, 39^ As these errors represent a standard route to drug-induced AKI and other serious events, such process gains are clinically relevant even when not all of the adjudicated ADEs have been captured. The substantial heterogeneity in early dosing trials, however, suggests that impact depends heavily on local thresholds, targeted drug classes, degree of EHR integration, and provider response to prompts. ^29– 32, 35^ We caution against over-interpreting the pooled magnitude. Although the random-effects point estimate favored CDSS (RR 1.76), the corresponding 95% prediction interval (0.75–4.14) spans from a modest adverse effect to a large benefit; formally, the expected effect in a new, exchangeable implementation cannot be assumed to be protective. Our inference that renal CDSS improve proximal medication-safety processes therefore rests not on the precision of this pooled estimate but on the convergence of two more stable observations: a consistent direction of effect across the majority of dosing/prescribing trials in the Best Evidence Synthesis (five of seven favoring CDSS), and the reproducible, low-heterogeneity signal for CKD documentation (I ^2^ = 0%). The dosing meta-analysis is best understood as showing that benefit is achievable but conditional on local implementation, rather than as a stable quantification of that benefit. Consistent with this, our configuration-based Evidence Map ( Figure 4) indicates that the densest evidence for process improvement clusters in interruptive, order-entry implementations, whereas clinical outcomes are reported far less consistently across configurations.

### 4.2 Why downstream clinical outcomes remain equivocal

By contrast, effects on renal function, cardiovascular events, and survival were inconsistent. A small number combined a CDSS with other interventions, such as practice facilitation or intensive management, and found positive eGFR trajectories and improved renal cortical blood flow, or less severe intradialytic hypotension, but negative hemoglobin control, CKD quality measures, and survival. ^33, 36, 38– 40, 42– 44, 48^ This discrepancy between relatively obvious benefits in proximal safety process measures and neutral downstream outcomes is not surprising given that trials were powered on process endpoints; the follow up periods were short; the CKD populations studied were heterogeneous with co-morbidities and competing risks; and those running concurrently other quality initiatives (even significant reductions in dosing error rates might equate to relatively minor absolute alterations in hard outcomes).

Figure 5 further confirms the fragility of the evidence base. By visualizing the network at the trial level, we observe that clinical-stage testing is restricted to a substantially smaller number of configurations than the raw trial counts might imply. This underscores an important interpretive risk: patient-level clinical conclusions would be extrapolated from an evidence base dominated by process-level data, and such extrapolation must be undertaken with caution.

### 4.3 Documentation improvements are plausible enablers but not substitutes for safety outcomes

CDSS also slightly increased EHR-documented CKD recognition in trials that focused on problem-list documentation and risk stratification; being labeled appropriately is a condition precedent to activating renal-dosing rules, monitoring protocols, or nephrology referral prompts. ^33, 34, 42, 44, 48^ The majority of studies considered transparent rule systems that capture dosing thresholds or contraindication-related information, whereas AI-enabled tools focused on higher-level predictive tasks, such as optimizing erythropoiesis-stimulating agent dosing or estimating cardio-renal risk, rather than broader formulary-wide dose checking. ^29– 32, 35, 36, 38– 40, 42, 48^ Randomized evidence suggests that AI-based systems are no less effective than usual care on process-level and physiological surrogate outcomes. To date, we have not seen any full publication of an RCT showing a clear benefit over a well-designed rule-based system in patient-centered safety. This is a crucial distinction. With this in mind, Table 7 reflects “evidence signals” that are configuration-specific, as well as ongoing reporting gaps that continue to enable us to ascertain whether a system’s success is due to its algorithm or to better workflow integration.

### 4.4 Implementation and governance determine whether safety potential is realized

Implementation and usability results provide insight into why the safety potential of CDSS is incompletely realized. Adherence of providers to the CDSS’s advice varied from very low to moderate. Two main and disadvantageous themes (alert fatigue, time pressure) as well as seven other recurring patterns in the qualitative findings (lack of training, weak technical integration, limited use or coordination with pharmacists, limited involvement in nephrologist consultations) were identified. ^29, 31– 33, 36, 37, 43– 46^ High rates of non-specific alarms can desensitize clinicians and add to alert-fatigue burden (e.g., hazard related to automation when critical warnings are missed), unclear attribution for responding to high-risk activations, and weak embedding within multidisciplinary care can further dampen effects on patient clinical outcomes. ^32, 36, 45, 47^ This study does not characterize CDSS as software. Still, as a socio-technical intervention, it draws attention to the fact that safety is as much about governance and workflow as it is about algorithmic accuracy. Accordingly, we treated delivery mode and workflow stage not as fixed background characteristics but as integral, modifiable components of the intervention itself. To put these findings into context, Extended data (Appendix 2: Trial-level mapping table) of the current study offers an in-depth examination of the trials included. By examining how this evidence was assembled, we aim to clarify what the current evidence base can and cannot support — an exercise that is particularly important given the pace of technological change in this field.

### 4.5 Implications for practice: positioning renal CDSS within quality and patient safety programs

To health systems and quality-improvement leaders, none of this proves that renal-dose CDSS raises survival. Current evidence suggests treating the thing as safety-critical infrastructure whose primary value in tertiary analyses may be preventing avoidable prescribing errors such as overdose of renally cleared agents, failure to adjust dosing down with falling eGFR, lapse in initiation of ACEi/ARB therapy for most indications other than AKI with HFrEF, or lag in being referred to nephrology. ^29– 32, 35, 38, 39, 45, 47^ Deployment should accompany medication safety initiatives in programs, including pharmacist-led review of high-risk prescriptions, standardized CKD order sets, and promoting drug-kidney education. A few simple metrics, the proportion of renally inappropriate orders, patterns of overrides for high-risk alerts, and serious medication-related events can recalibrate thresholds, eliminate low-value rules, and pinpoint services calling for support. In contrast, alert design should focus on high-risk scenarios with straightforward recommendations to incorporate into the workflow of ordering. ^29, 31– 33, 36, 37, 39, 40, 42– 46, 48^ Our configuration-based synthesis further suggests that implementation decisions (e.g., interruptive alerts at order entry vs non-interruptive dashboards) should be considered part of the intervention itself, because they condition adoption and the plausibility of achieving clinically meaningful downstream impact.

### 4.6 Future research agenda

Three guiding considerations arise from the existing evidence. ^29– 32, 35, 36, 38, 40, 42, 48^ Pragmatic (sufficiently powered, head-to-head RCT) comparisons between AI-CDSS and mature rule-based systems are required to establish whether advanced prediction and personalization provide incremental safety or cost-effectiveness gains beyond those achieved with effectively implemented rules. Second, a CKD-CDSS core outcome set, which includes measurement of renal-dose accuracy: EHR-documented CKD recognition; nephrology referral; blood pressure control; and important patient safety outcomes such as acute kidney injury (AKI), serious ADRs, or MHRs, improves comparability of studies. Third, new trials should adhere to such frameworks as CONSORT-AI for designing more complex interventions that establish implementation parameters as primary outcomes driven by mediation analyses linking prescription-process gains to clinical endpoints, with consideration of transparency, bias (eg, endogenously impacting trained vs nontrained sites), data drift crossover, and Equity in AI-CDSS models. To address the recurring gaps in the evidence observed ( Table 7, Extended data in Appendix 2: Trial-level mapping table), future trials may need to include better reporting of outcomes. This encompasses detailed trigger logic, the alerting delivery mechanism, and levels of severity, as well as a mindset toward the reporting of override data. And indeed, the slogan that ownership lineage and governance for model updates are a substitute for what is wanted has proven to be very true. These are the factors that determine whether a study’s data can contribute to interpretation or be included in subsequent meta-analyses.

### 4.7 Strengths and limitations

This systematic review was reported according to the PRISMA guidelines, was prospectively registered in PROSPERO, and included only randomized controlled trials, thereby improving internal validity compared with mixed-design reviews. When outcome-specific RoB 2 evaluations were applied and random-effects models with fixed-effect sensitivity analyses fitted, there was evidence that process outcomes differed from clinical endpoints and a physical apparent pattern in time; i.e., earlier dosing studies yielded larger, although more heterogeneous effects, while more recent EHR-integrated trials gave rise to smaller but less diverse gains in CKD awareness and associated processes. Another strength is the structured nature of the synthesis and the visualization of the architecture of the evidence at the trial level ( Figure 4 and Figure 5, Table 7). More than just creating identifiable pooled effects, these methods provide a nuanced tool for navigating research in which heterogeneity is so vast. They guard against the interpretive myopia that would allow our calculations to be reported but not properly taken into account in their specific context.

It is important limitations must also be recognized. There was significant heterogeneity across many of the most important outcomes, driven by differences in outcome definitions and trial design; only a proportion of eligible trials contributed to each meta-analysis. In particular settings, there were too few studies with effect estimates to allow informative subgroup analyses or exploration of publication bias. Key domains were at risk of “some concern” or “high” bias in several studies, and the majority of studies took place in high-income countries with well-established EHR systems. Few trials explicitly quantified adjudicated adverse drug events and other patient-centred safety measures, so estimates of harm reductions continue to be based largely on the premise that improvements in prescribing practice translate into less harm. ^29– 32, 35, 38^ We acknowledge, however, that the most consistent signal is around improved renal dosing and related prescribing practices. Finally, because trial reporting of configuration and implementation details was frequently incomplete, the Evidence Map and network visualization necessarily reflect what was reported; Table S2 is therefore provided to transparently document reporting presence/absence and to guide more reproducible future evaluations.

Generalizability beyond high-income, EHR-mature settings. Twelve of the twenty included trials were conducted in the United States, and almost all were set in health systems with mature, integrated electronic health records and computerized provider order entry. This concentration constrains transportability in three concrete ways. First, the dominant effective configuration we identified — interruptive, order-entry alerts embedded in an EHR/CPOE — presupposes infrastructure that is unavailable or only partially implemented in many low- and middle-income countries (LMICs), where prescribing may remain paper-based or split across non-interoperable systems; there, the relevant evidence would come from standalone or mobile decision aids, a configuration represented here by only a handful of small trials and never tested against hard clinical endpoints. Second, rule content is calibrated to local formularies, renal-function estimating equations, and reference thresholds; a dosing rule validated against one formulary cannot be assumed to transfer to a different drug-availability, generic-substitution, or guideline environment without local re-derivation and revalidation. Third, the workforce and workflow enablers that plausibly drive the observed benefit — clinical-pharmacist review, nephrology back-up, and dedicated implementation support — vary markedly across health systems. Before renal CDSS can be regarded as a globally applicable safety tool, pragmatic trials are needed in resource-variable and LMIC settings, evaluating implementation-appropriate configurations (including offline-capable mobile tools), locally validated rule content, and patient-centred safety outcomes rather than process surrogates alone. Until such evidence exists, the present findings should be read as applying primarily to well-resourced, EHR-integrated environments.

Target population and its heterogeneity. Our eligibility criterion — adults with CKD or at demonstrable risk of CKD-related prescribing error — was deliberately pragmatic, reflecting how renal-dosing CDSS are actually triggered (by an eGFR/creatinine threshold at prescribing) rather than by a chronicity-defined diagnosis. We acknowledge that this criterion spans clinically distinct populations — acute kidney injury, early CKD (stages 1–2), advanced CKD (stages 4–5) approaching dialysis, and prevalent dialysis — each with different baseline risk and prescribing-error profiles. Supplementary Table 9 shows that the pooled dosing trials predominantly enrolled mixed “renal impairment” populations defined by a laboratory threshold rather than by CKD stage, whereas dialysis-specific and screening/recognition trials targeted narrower groups. Given the small number of trials per outcome, subgroup and meta-regression by population stratum were not estimable, so we treat population mix as a further, unquantified source of the clinical heterogeneity captured by our I ^2^ values and prediction intervals.

In particular, for the primary dosing outcome the prediction interval crossed unity, so the pooled estimate constrains what can be claimed about transportable effectiveness and should not be read as evidence of assured benefit in any specific setting.

## 5. Conclusion

Rule-based and AI-driven CDSS have successfully augmented proximal drug safety in CKD by increasing the accuracy of dosing and prescribing practices. However, it has been challenging to demonstrate the link between these systems and long-term clinical endpoints, such as renal function, CVD outcomes, or overall mortality. One important conclusion emerges from this heterogeneous body of studies. There is no inherent property that determines whether a CDSS will be effective; instead, it is contingent on the manner in which that system interacts with clinical work processes. Such findings indicate a new paradigm, from CDSS as a complement to CDSS as a safety-critical infrastructure. The underpinning of safe systems that are both safe and trustworthy requires a requisite enabling environment, inclusive of regulation, a human-centered design approach, and adherence to long-term monitoring. The focus of science should thus shift to more systematic head-to-head comparisons of AI vs. traditional rule-based systems. Sufficient data quality that adds to global-synthesis projects implies high study power and full reporting transparency; these are fundamental hallmarks of good research, which should not have to be traded off for the “opportunity” to take part in larger studies.

## Declaration of generative AI and AI-assisted technologies in the manuscript preparation process

During the preparation of this paper, the authors used AI-assisted language tools (ChatGPT and Grammarly) to help improve English grammar and phrasing in these typescripts. All content of the scientific manuscripts (study design, data extraction and analysis, interpretation, and conclusions) has been developed by the authors. No AI tool produced any original scientific content. The authors thoroughly examined all AI-generated text and fully accept responsibility for the final manuscript content.

## Data Availability

Zenodo: PRISMA 2020 Checklist v2 and PRISMA flow diagram, Appendix for “Improving Medication Safety in Chronic Kidney Disease Using Rule-Based and Artificial Intelligence–Based Clinical Decision Support Systems: A Systematic Review of Randomized Controlled Trials”. Working DOI: https://doi.org/10.5281/zenodo.18678563. License: CC0 1.0. ^49^ Data screening, extraction, and manuscript preparation were performed using Microsoft Office (Word/Excel) via an official student-licensed subscription provided by Universitas Indonesia. Study screening and selection were managed in Covidence (trial version). Meta-analysis was conducted using RevMan 5.2 (trial version; accessed September 2025). Network mapping and visualization were performed using Cytoscape (version 3.10.4). Figure 4 was created using Canva under an education/school license. Literature searches were conducted using Scopus, ScienceDirect, and ProQuest accessed through Universitas Indonesia’s institutional subscriptions. PubMed was accessed free of charge, and full-text access for paywalled articles was obtained where available through Universitas Indonesia’s institutional licenses. This systematic review is reported in accordance with the PRISMA 2020 statement. The PRISMA flow diagram is presented in Figure 1. The completed PRISMA 2020 checklist is available as Extended data ( https://doi.org/10.5281/zenodo.18678563). ^49^ License: CC0 1.0.
