# Supplementary material for: Improving Medication Safety in Chronic Kidney Disease Using Rule-Based and Artificial Intelligence–Based Clinical Decision Support Systems: A Systematic Review of Randomized Controlled Trials
Source: F1000Res. 2026 Aug 28;15:532. Originally published 2026 Apr 16. [Version 2] doi: 10.12688/f1000research.178438.2 (PMC13583346; doi:10.12688/f1000research.178438.2)
Supplement: Peer review history

# Reviewer report: Reviewer response for version 2

**DOI:** 10.5256/f1000research.208963.r515420

**Published:** 2026-09-16

**Authors:** Khulud Alsulami

**Affiliations:** 1 King Abdulaziz City for Science And Technology, Riyadh, Riyadh Province, Saudi Arabia

Thank you. The amendments substantively answer the concerns listed in the previous comment; however, two minor corrections are required:

 1. The abstract and Section 3.4.1 still *lead* with "CDSS significantly improved appropriate renal dosing... RR 1.76... p=.01" as the topic sentence, with the prediction-interval caveat following afterwards. Given that the stated goal of the revision was that "the primary conclusion no longer rests on the pooled dosing estimate," the framing at first mention is still somewhat headline-driven before the caveat lands.

 2. Table.3 ("Characteristics of the included studies") still lists "Sperl-Hillen, 2023" in both the label and Year column, even though the reference list entry and every other in-text citation now correctly say 2024.

Are the rationale for, and objectives of, the Systematic Review clearly stated?

Yes

Is the statistical analysis and its interpretation appropriate?

I cannot comment. A qualified statistician is required.

If this is a Living Systematic Review, is the ‘living’ method appropriate and is the search schedule clearly defined and justified? (‘Living Systematic Review’ or a variation of this term should be included in the title.)

Not applicable

Are sufficient details of the methods and analysis provided to allow replication by others?

Yes

Are the conclusions drawn adequately supported by the results presented in the review?

Yes

Reviewer Expertise:

cardiovascular research, Single-cell technologies

I confirm that I have read this submission and believe that I have an appropriate level of expertise to confirm that it is of an acceptable scientific standard.

# Reviewer report: Reviewer response for version 2

**DOI:** 10.5256/f1000research.208963.r516535

**Published:** 2026-09-16

**Authors:** Sunandar Ihsan

**Affiliations:** 1 Universitas Halu Oleo, Kendari, Indonesia

Here are a few points to consider.

 1. Abbreviations are defined on first mention (CDSS, CPOE, GFR, BP/CV), or the defined abbreviation for the first time is used (risk of bias)

 2. Health professionals or healthcare professionals (HCPs)? (in the Discussion section).

 Clarification is needed regarding a few sentences.

 3. In accordance with kidney function guidelines (the first row of the second column) Which guidelines are being referred to? Please clarify.

 4. In the Results section: a total of 20 trials met inclusion criteria, and 6 of them underwent quantitative analysis. However, the PRISMA table seems to imply that 26 studies were to be analyzed. Please clarify.

 5. In the results section (mortality events), please clarify how hospital admission is considered an adverse event, along with other significant adverse events.

 6. Section Usability and provider acceptance: clinician acceptance of CDSS was moderate and varied considerably. What does "varied" mean in this context? Please clarify.

 7. Section Guideline Adherence and clinical process measures: Medical security or safety? Please clarify.

Are the rationale for, and objectives of, the Systematic Review clearly stated?

Yes

Is the statistical analysis and its interpretation appropriate?

Yes

If this is a Living Systematic Review, is the ‘living’ method appropriate and is the search schedule clearly defined and justified? (‘Living Systematic Review’ or a variation of this term should be included in the title.)

Yes

Are sufficient details of the methods and analysis provided to allow replication by others?

Yes

Are the conclusions drawn adequately supported by the results presented in the review?

Yes

Reviewer Expertise:

Clinical Pharmacy, Drug Evaluation Study, Drug Compatibility and Stability

I confirm that I have read this submission and believe that I have an appropriate level of expertise to confirm that it is of an acceptable scientific standard.

# Reviewer report: Reviewer response for version 1

**DOI:** 10.5256/f1000research.196822.r492569

**Published:** 2026-07-28

**Authors:** Khulud Alsulami

**Affiliations:** 1 King Abdulaziz City for Science And Technology, Riyadh, Riyadh Province, Saudi Arabia

This is a clinically relevant systematic review addressing an important gap in nephrology informatics. The authors demonstrate methodological awareness and make laudable attempts to address heterogeneity through supplementary synthesis frameworks. However, there are several substantive limitations, some were acknowledged and some were not, constrain the conclusions that can be drawn. 

 1. Critical Underpowering of the Meta-Analysis

 This is the review's most significant structural weakness. Of 20 included RCTs, only 6 contributed to meta-analysis, and the primary dosing outcome pools just 4 studies. With I² = 97%, the random-effects pooled estimate has a prediction interval of 0.75–4.14, which crosses unity and encompasses both harm and large benefit. This interval is so wide as to provide almost no useful precision. The authors acknowledge this but do not adequately address what this means for their headline conclusion.

 2. The Exclusion of 14 Studies from Meta-Analysis Is Inadequately Explained

 The paper does not provide a sufficiently detailed account of why 14 of 20 eligible RCTs could not contribute to quantitative synthesis. A supplementary table documenting, for each excluded-from-meta-analysis study, the specific reason for exclusion from pooling would substantially strengthen transparency.

 3. Geographic and Healthcare System Homogeneity

 Fifteen of the 20 included trials were conducted in the United States, and nearly all took place in high-income settings with established EHR infrastructure. The authors acknowledge this in limitations but do not adequately explore its implications. A more sustained discussion of this gap, and of what research is needed before renal CDSS can be considered a global tool, would materially strengthen the paper's contribution.

 4. The "CKD at Risk" Population Is Ambiguous

 The inclusion criteria specify "adults with CKD or at risk of CKD-related prescribing errors." The latter category is highly heterogeneous and potentially includes almost any hospitalized patient with reduced renal function, regardless of CKD stage or chronicity. Studies enrolling patients with acute kidney injury, patients with CKD stages 1–2, and patients with stage 4–5 CKD facing imminent dialysis represent fundamentally different populations with different prescribing risk profiles. The review does not systematically stratify or account for this population heterogeneity in its synthesis.

 5. Stylistic Inconsistency

 The declaration of AI-assisted writing tools (ChatGPT and Grammarly) is appreciated, though some passages in the discussion show stylistic inconsistency that suggests incomplete editing. The phrase "very Big Risk" in section 4.2 appears to be an artifact of informal drafting. Several sentences in the discussion are syntactically awkward in ways that compromise precision (e.g., "This transition in perspective is also why we deviated from treating transfer modes and workflow kinetics as frozen 'background' variables"). These should be addressed in revision.

Are the rationale for, and objectives of, the Systematic Review clearly stated?

Yes

Is the statistical analysis and its interpretation appropriate?

I cannot comment. A qualified statistician is required.

If this is a Living Systematic Review, is the ‘living’ method appropriate and is the search schedule clearly defined and justified? (‘Living Systematic Review’ or a variation of this term should be included in the title.)

Not applicable

Are sufficient details of the methods and analysis provided to allow replication by others?

Yes

Are the conclusions drawn adequately supported by the results presented in the review?

Yes

Reviewer Expertise:

cardiovascular research, Single-cell technologies

I confirm that I have read this submission and believe that I have an appropriate level of expertise to confirm that it is of an acceptable scientific standard, however I have significant reservations, as outlined above.

# Response

**Published:** 2026-08-19

**Authors:** Asrul Ismail

**Affiliations:** Pharmacy, University of Indonesia Center for Clinical Epidemiology and Evidence Based Medicine, Jakarta, Special Capital Region of Jakarta, Indonesia

We thank Dr. Alsulami for a careful and constructive review, and for approving the article with reservations. The five points raised are well taken; each has been addressed through revisions to the text and two new tables (Tables 8 and 9), with no change to the underlying data or analyses. Below we respond to each comment in turn — quoting the comment, giving our response, and listing the specific changes. Section and reference numbers refer to the revised manuscript.

**1. Critical underpowering of the meta-analysis**

**Reviewer comment:** "Of 20 included RCTs, only 6 contributed to meta-analysis, and the primary dosing outcome pools just 4 studies. With I² = 97%, the random-effects pooled estimate has a prediction interval of 0.75–4.14, which crosses unity and encompasses both harm and large benefit …" The authors acknowledge this but do not adequately address what this means for their headline conclusion.

**Response : **We agree, and we accept the precise statistical point: a prediction interval spanning 0.75–4.14 formally implies that, for a new exchangeable implementation, a protective effect cannot be assured and a null or adverse effect cannot be excluded. We have revised the manuscript so that our headline inference no longer rests on the magnitude of the pooled dosing estimate. Instead, it rests on the convergence of two more stable observations: a consistent direction of effect across the majority of dosing/prescribing trials in the Best Evidence Synthesis (five of seven favoring CDSS), and the reproducible, zero-heterogeneity signal for CKD documentation (I² = 0%). The dosing meta-analysis is now framed as showing that benefit is achievable but conditional on local implementation, rather than as a stable quantification of that benefit.

**Changes: ** (i) The Abstract (Results) now states the prediction interval explicitly and reframes the pooled RR as a context-dependent average; (ii) a paragraph added to Section 4.1 makes the interpretive ceiling explicit; (iii) a sentence added to Section 4.7 links this imprecision to the limitations. The revised Abstract text reads:

 "Pooled across four trials, CDSS improved appropriate renal dosing (RR 1.76; 95% CI 1.13–2.74), but heterogeneity was extreme (I² = 97%) and the 95% prediction interval (0.75–4.14) crossed the null; the pooled estimate is therefore a context-dependent average rather than a transportable effect, and benefit cannot be assured in a new setting. A consistent direction of effect favoring CDSS came instead from the Best Evidence Synthesis."

**2. The exclusion of 14 studies from meta-analysis is inadequately explained**

**Reviewer comment:** The paper does not provide a sufficiently detailed account of why 14 of 20 eligible RCTs could not contribute to quantitative synthesis. A supplementary table documenting, for each excluded-from-meta-analysis study, the specific reason for exclusion from pooling would substantially strengthen transparency.

**Response:** We agree, and we have made the reason explicit. It is a single methodological one: quantitative pooling requires two or more trials reporting an outcome with a commensurable operational definition, denominator, and effect metric. Only two outcome families met this criterion — appropriate renal dosing (four trials) and EHR-documented CKD recognition (two trials). For the dosing pool we harmonized all four trials to one construct (proportion of medication instances/orders appropriately dosed), deriving 'appropriate' counts by arithmetic complement where a trial reported only an 'inappropriate/excessive dosing' proportion, without altering denominators (Table 2). No such harmonization was possible for the remaining domains.

 The blood-pressure domain illustrates the constraint. Although two trials reported BP outcomes, they were not the same outcome: Peralta (2020) reported sustained BP control (<140/90 mmHg) as a patient-level proportion, whereas Samal (2024) reported a continuous change in systolic BP (−14.6 vs −11.7 mmHg). These differ not only in threshold framing but in data type — a risk ratio versus a mean difference — and cannot be combined. A parallel problem affects the ACEi/ARB-initiation and CKD-recognition domains, where trials used non-equivalent denominators.

**Changes: ** We have added Table 8 (shown below in compact form; the full table, with the outcome(s) and the effect as reported for each trial, appears in the manuscript), listing each of the 14 included-but-not-pooled trials with the specific reason it was non-commensurable. We also note that several of these trials had individually clear definitions and denominators; they were excluded from pooling solely because no second trial measured an identical outcome — underscoring that commensurability, not study quality, was the binding constraint. Cross-references to Table 8 were added to the Methods (Section 2.6) and Results (Section 3.1), with a short introductory paragraph preceding the table.

 The full version of this appears as Table 8 in the revised article; for the reviewer's convenience, the same information is condensed below. Each trial is followed by a code and the specific reason it could not be pooled. Codes: A = unique outcome with no pooling partner; B = same domain but a different definition, denominator, or metric; C = not a comparative CDSS implementation study.

 Abdel-Kader (2011) [48] — code A/B — Referral and testing outcomes, not the CKD-diagnosis documentation of the recognition pair.

 Alhodaib (2020) [37] — code A — Clinician-level decision-accuracy score from simulated scenarios; a unique construct.

 Carroll (2018) [42] — code B — Overlaps the recognition construct but reports no extractable effect size.

 Chen (2022) [39] — code A — Nutrition and quality-of-life domain, outside the pooled safety constructs.

 Erler (2012) [35] — code B — Patient-level "any overdose" (OR), not order-level appropriateness (RR).

 Januzzi (2025) [40] — code C — Prediction-model development only; no comparative CDSS process outcome.

 Lim (2025) [36] — code A — ESA/anemia control; model-selection design; per-test denominators not extractable.

 Locatelli (2009) [45] — code A — Dialysis-anemia target attainment; a unique domain; denominators not reported.

 Mancini (2007) [38] — code A — Session-level crossover design, incompatible with parallel-group pooling.

 Patzer (2018) [46] — code A — Transplant-education domain, outside the pooled safety constructs.

 Peralta (2020a) [44] — code B — Recognition denominator and RAS new-use definition differ from the poolable outcomes.

 Samal (2022) [43] — code B — Monitoring and referral construct; denominator derivation not fully specified.

 Samal (2024) [41] — code B — Continuous SBP change (mean difference) versus dichotomous BP control; differing ACEi/ARB denominator.

 Sequist (2018) [47] — code B — Risk-stratified referral and testing; a different construct and denominator.

**3. Geographic and healthcare-system homogeneity**

**Reviewer comment:** Fifteen of the 20 included trials were conducted in the United States, and nearly all took place in high-income settings with established EHR infrastructure … A more sustained discussion of this gap, and of what research is needed before renal CDSS can be considered a global tool, would materially strengthen the paper's contribution.

**Response:** We are grateful for this point and have expanded it into a dedicated discussion of transportability. On re-checking Table 3, twelve of the twenty trials were conducted in the United States; the remaining eight were set in the United Kingdom, Germany, Canada, Italy, Taiwan, China, a nine-country Central/Eastern European cluster, and a 34-country trial. The reviewer's underlying concern is entirely valid nonetheless: the large majority of trials were conducted in high-income, EHR-mature health systems, and this constrains generalizability in concrete ways that we now spell out.

**Changes:** A new paragraph ('Generalizability beyond high-income, EHR-mature settings') was added to Section 4.7, explaining three specific limits on transportability — (a) the dominant effective configuration (interruptive order-entry alerts in an EHR/CPOE) presupposes infrastructure often unavailable in low- and middle-income countries, where the relevant evidence would instead come from standalone/mobile tools tested here only in small trials and never against hard endpoints; (b) rule content is calibrated to local formularies, renal-function equations, and thresholds, and cannot be assumed to transfer without local re-derivation and revalidation; and (c) the workforce/workflow enablers plausibly driving benefit (clinical-pharmacist review, nephrology back-up, implementation support) vary markedly across systems. We also specify the pragmatic, resource-variable trials with implementation-appropriate configurations and patient-centered outcomes that are needed before renal CDSS can be regarded as globally applicable.

**4. The "CKD at risk" population is ambiguous**

**Reviewer comment:** Studies enrolling patients with acute kidney injury, patients with CKD stages 1–2, and patients with stage 4–5 CKD facing imminent dialysis represent fundamentally different populations with different prescribing risk profiles. The review does not systematically stratify or account for this population heterogeneity in its synthesis.

**Response: **This is a fair and important point. Our eligibility criterion was deliberately pragmatic because renal-dosing CDSS are triggered by reduced kidney function at the point of prescribing (an eGFR/creatinine threshold) rather than by a chronicity-defined diagnosis; restricting to formally staged chronic CKD would have excluded the order-entry dosing trials that constitute the core of the medication-safety evidence (e.g., long-term-care and emergency-department renal-insufficiency populations). We now make the resulting heterogeneity explicit.

**Changes:** (i) A clarifying passage was added to the Discussion (Section 4.7) acknowledging that the criterion spans clinically distinct populations (AKI, CKD G1–2, advanced CKD G4–5, and prevalent dialysis). (ii) Table 9 post-hoc characterizes every included trial by its enrolled renal population. It shows a clear pattern that directly addresses the reviewer's concern: the four pooled appropriate-dosing trials all defined their population by a creatinine-clearance/eGFR threshold (mixed 'renal impairment', often inpatient/ED/long-term-care) rather than by CKD stage, whereas the two pooled recognition trials enrolled staged CKD G3–G4 primary-care populations, and a distinct dialysis cluster was never pooled. (iii) Because trials per outcome were few, formal subgroup or meta-regression by population stratum was not estimable; we therefore state that population mix is an additional, unquantified source of the clinical heterogeneity reflected in our I² values and prediction intervals, and flag population-specific effect estimation as a priority for future trials.

**5. Stylistic inconsistency**

**Reviewer comment: **The phrase 'very Big Risk' in section 4.2 appears to be an artifact of informal drafting. Several sentences in the discussion are syntactically awkward in ways that compromise precision … These should be addressed in revision.

**Response: **We are grateful for this careful reading and have copy-edited the Discussion in full, and standardized spelling to US English throughout the manuscript. The specific phrases noted have been rewritten, and we corrected several further issues found during this pass (listed in the next section).

**Changes:** Representative rewrites: the sentence beginning 'This raises a Very Big Risk…' (§4.2) is replaced by 'This underscores an important interpretive risk: patient-level clinical conclusions would be extrapolated from an evidence base dominated by process-level data, and such extrapolation must be undertaken with caution.' The 'frozen background variables' sentence (§4.4) is replaced by 'Accordingly, we treated delivery mode and workflow stage not as fixed background characteristics but as integral, modifiable components of the intervention itself.' Verbatim before/after text for every edit is provided in the accompanying Revision Schedule.

**Additional corrections made during revision**

 During this revision we identified and corrected the following, in the interest of accuracy and internal consistency:

 Erler effect size (3.4.2). The overdose result was corrected to OR 0.46 (95% CI 0.26–0.82), p = 0.008. The previously printed 'OR 0.45; p < 0.001' is inconsistent with the reported confidence interval (which corresponds to p ≈ 0.008) and with our extraction records.

 Citation and year for Sperl-Hillen (3.5.1). The in-text reference superscript was corrected from 41 to 33 (reference 41 is Samal 2024), and the publication year from 2023 to 2024 (Kidney Med. 2024; 6(3):100777), consistent with the reference list.

 Sub-section numbering (Discussion). The Discussion contained two sections numbered 4.4 and no 4.5. 'Implications for practice: positioning renal CDSS within quality and patient safety programs' has been renumbered to 4.5, restoring a continuous 4.1–4.7 sequence.

 Terminology and attribution. The word 'antigens' in the Introduction (an evident error) was removed. In 3.5.2, 'our randomized hemodialysis trial' was corrected to 'the single randomized hemodialysis trial (Lim 2025)', which is an included trial, not an author-conducted study.

 Heterogeneity statistic (3.4.1 vs Table 4). The Cochran Q for appropriate dosing, previously reported as χ² = 72.66 in 3.4.1 and the forest-plot footnote but as 76.35 in Table 4, has been reconciled to χ² = 72.66 throughout (I² = 97% unchanged).

 New tables. Table 8 (trials not entered into meta-analysis, with reasons) and Table 9 (target renal population by trial) have been added to the manuscript body. Because F1000Research places no limit on the number of tables, these are included directly in the article rather than as external supplementary files; the Extended data (Zenodo) deposit is therefore unchanged.

 We believe these revisions address each of the reviewer's reservations directly, and we are grateful for the improvements they have prompted.
